# Enhanced Nanofiltration Process of Thin Film Composite Membrane Using Dodecyl Phenol Ethoxylate and Oleic Acid Ethoxylate for Oilfield Calcite Scale Control

**DOI:** 10.3390/membranes11110855

**Published:** 2021-11-04

**Authors:** Saedah R. Al-Mhyawi, Mahmoud F. Mubarak, Rasha Hosny, Manal Amine, Omnia H. Abdelraheem, M. A. Zayed, Ahmed H. Ragab, Abeer El Shahawy

**Affiliations:** 1 Department of Chemistry, College of Science, University of Jeddah, Jeddah 21419, Saudi Arabia; sral-mhyawi@uj.edu.sa; 2 Petroleum Application Department, Egyptian Petroleum Research Institute (EPRI), Nasr City, Cairo 11727, Egypt; 3 Production Department, Egyptian Petroleum Research Institute (EPRI), Nasr City, Cairo 11727, Egypt; dr.rashahosny@yahoo.com or; 4 Processes Design & Development Department, Egyptian Petroleum Research Institute (EPRI), Nasr City, Cairo 11727, Egypt; manal_amin2000@yahoo.com; 5 Engineering Sciences Department, Faculty of Engineering, Beni-Suef University, Beni-Suef 62511, Egypt; omnia_hassan55@yahoo.com; 6 Chemistry Department, Faculty of Science, Cairo University, Giza 12613, Egypt; Zayed@sci.cu.edu.eg; 7 Department of Chemistry, Faculty of Science, King Khalid University, Abha 62224, Saudi Arabia; ahrejab@kku.edu.sa; 8 Department of Civil Engineering, Faculty of Engineering, Suez Canal University, Ismailia 41522, Egypt

**Keywords:** produced water treatment, oilfield calcite scale control, scales precipitation, scale inhibitors, non-ionic surfactants, alkylethoxylate, nanofiltration membrane

## Abstract

This research studied the enhancing effect on the nanofiltration composite (TFCNF) membrane of two non-ionic surfactants on a thin-film composite nanofiltration membrane (TFCNF) for calcite scale (CaCO_3_) inhibition in oilfield application to develop a multifunctional filtration system: nanofiltration, antiscalant, and scale inhibitors. The effectiveness of dodecyl phenol ethoxylate (DPE) and oleic acid ethoxylate (OAE) as novel scale inhibitors were studied using the dynamic method. Scaling tests on the membrane were performed to measure the scaling of the inhibited membrane with and without scale inhibitors for salt rejection, permeability, and flux decline. The results revealed that the TFCNF membrane flux decline was improved in the presence of scale inhibitors from 22% to about 15%. The rejection of the membrane scales increases from 72% for blank membranes, reaching 97.2% and 88% for both DPE and OAE, respectively. These confirmed that scale inhibitor DPE had superior anti-scaling properties against calcite deposits on TFCNF membranes. Inhibited scaled TFCNF membrane was characterized using environmental scanning electron (ESEM), FTIR, and XRD techniques. The results of the prepared TFCNF membrane extensively scaled by the calcite deposits were correlated to its morphology.

## 1. Introduction

Scale formation of produced water [1,2] generated during oil or natural gas exploitation may occur due to the incompatibility between the formation water and the injected seawater [3]. The result deposits adhere near the surfaces of the well-producing tubing, perforations, tubing, sub-sea equipment, which accumulates over time and causes problems in reservoirs, topside facilities, and impairs oil production [4]. Calcium carbonates, “calcite”, are scale-formed in the oil and gas industry, causing high costs and significantly reducing production rates and equipment damages [5].

Membrane separation is categorized as advanced wastewater treatment technology and has the advantages of high operating efficiency, easy scale-up, and less space demand [6,7,8]. Nanofiltration (NF) has been assigned as an efficient water treatment techniquebecause of its ability to treat a large variety of waters. As the NF membrane recovery percentage increases, the concentration of these sparingly soluble minerals salts also increases in the membrane, resulting in salt saturation on the membrane causing inorganic scaling [9,10]. Consequently, scales decrease membrane permeate flux and reduce life [11]. As a result, the consumption of scale inhibitors ensures the stability and duration of the system; consequently, much attention has been paid to scale inhibitor research [12,13,14,15]. Therefore, the selected antiscalants in specific applications have different capabilities for inhibiting scales in the NF membrane. The scale inhibitors holdup the crystal formation through slowing induction time, leading to a delay of crystal growth [16].

In PW treatment, scale inhibitors decrease scale residues on the membrane surface in a membrane-operated water treatment plant [17,18]. Many antiscalants fight to scale and fouling by attaching to the contaminants in the water, thereby inhibiting it from depositing on the membrane surface [19,20]. These contaminants are finally discarded in the drain of the NF [21]. Theantiscalants are available with variable chemistries based on phosphates, phosphonates, maleic, acrylates, and carboxylic acids [22,23].

Alkylphenol ethoxylate (APE) is one of the most widely used classes of surfactants.APEs are soluble in water and assist in the dispersion of dirt and grease from stained surfaces into water. APEs are essential to many industrial applications such astextiles, paper, coatings, plastics, pesticides, lubricants, and fuels [24]. Synthetic surfactants differ significantly in structure but are mainly composed of alkyl or alkylphenol groups joined to an anionic or non-ionic hydrophilic moiety. These compounds are generally safe and eco-friendly. Besides, they do not lead to pollution problems because they are subjected to biodegradation by micro-organisms existing in soils and surface waters [25].

The objective of this paper is to improve water treatment via decreasing calcite scale on TFCNF membranes surface. Therefore, in this research, two new scale inhibitors, dodecyl phenol ethoxylate(DPE) and oleic acid ethoxylate (OAE),were prepared and investigated through the spectroscopic techniques FTIR and HNMR. The role of DPE and OAE in calcite scaling control was evaluated depending on the change of permeate flux decline compared to that of the bared TFCNF membrane. The PVDF polymer is selected and used due to its following advantages; a high porosity and high water permeability, high chemical stability than polyvinyl chloride or polyethersulfone when it is used as a support, much lower layer thickness than other polymers, which saves in the amount used, enhances salt scales rejection without causing the injury of inner pores structures during water desalination processing, easy to recycle the used membrane, adding a new economic effect of using PVDF as membrane support.Scaled inhibited TFCNF membrane characterization via ESEM, FTIR, and XRD analysis were investigated. Moreover, the membrane scaling control mechanism in DPE and OAE was illustrated using different calcite solution concentrations.

## 2. Materials and Experimental

### 2.1. Materials

For the preparation of scale inhibitors, an ethylene oxide cylinder with a valve was obtained from the Eastern Company for Industrial Gases, Egypt; Dodecyl phenol (commercial grade) was purchased from Prolabo, Oleic acid (99%, from Merck-Schuchardt), and potassium hydroxide from Aldrich). Sodium carbonate (Na_2_CO_3_) and calcium chloride (CaCl_2_) were purchased from Aldrich. The solution pHwas adjusted by using NaOH and HCl solution. For membrane preparation, dimethyl-acetamide (DMAc), dichloromethane (DCM), chloroform, n-butanol, octylamine (OA), octadecylamine (ODA), Poly-vinylidene fluoride (PVDF) with molecular weight equal to 5.34 × 105 g.mol^−1^, hydrazine monohydrate (80 vol% in H_2_O) and phosphoric acid were obtained from Sigma Aldrich (Nottingham, UK).

### 2.2. Synthetic Produced Water

Different concentrations of Ca^2+^ and HCO_3_^−^ were prepared using CaCl_2_ and NaHCO_3_, as illustrated in Table 1. The synthetic calcite brine symbolizes the oilfield-produced water with a similar salinity, ionic composition, and pH value. Brine is synthesized by dissolving inorganic salts in deionized water. These CaCl_2_ and NaHCO_3_ solutions were prepared individually by weighing salts and dissolving them in deionized water. Before the experiment runs, brine is diluted 1:1 and filtered through filter paper (0.45 µm). NaOH and HCl solution was used for each test to adjust the pH = 7.81. Before being mixed, CaCl_2_ and NaHCO_3_ solutions were allowed to equilibrate with carbon dioxide in the atmosphere for 24 h.

### 2.3. Scale Inhibitors Preparation

**Figure 1:**
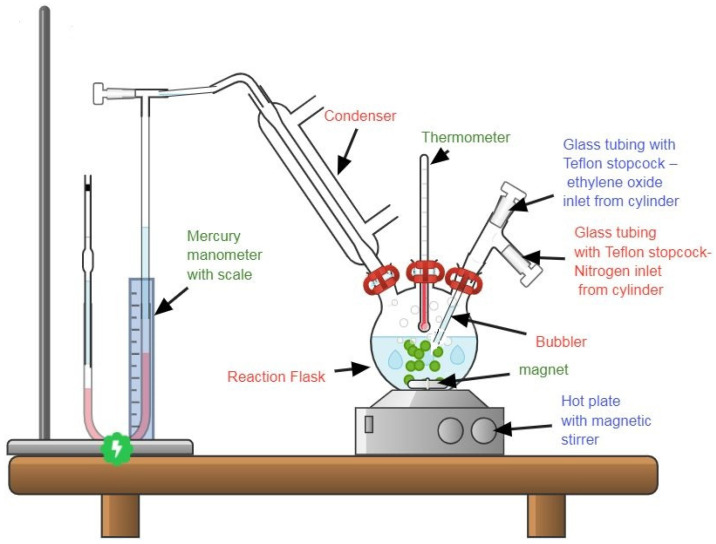
Batch laboratory-scale unit for ethoxylation reaction.

**Figure 2:**
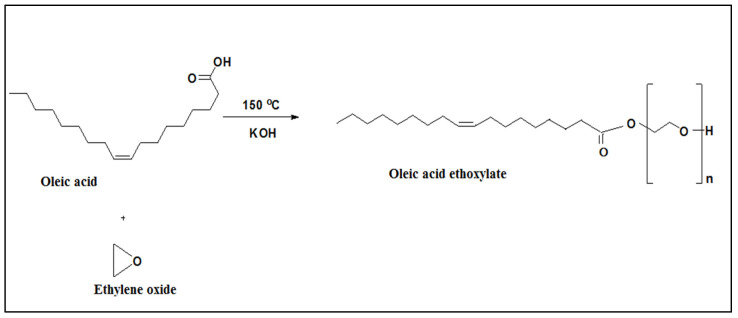
Synthesis approach for the preparation ofDodecyl phenol ethoxylate.

**Figure 3:**
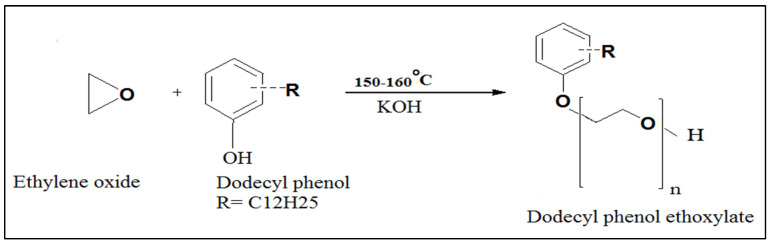
Synthesis route of oleic acid ethoxylate.

**Figure 4:**
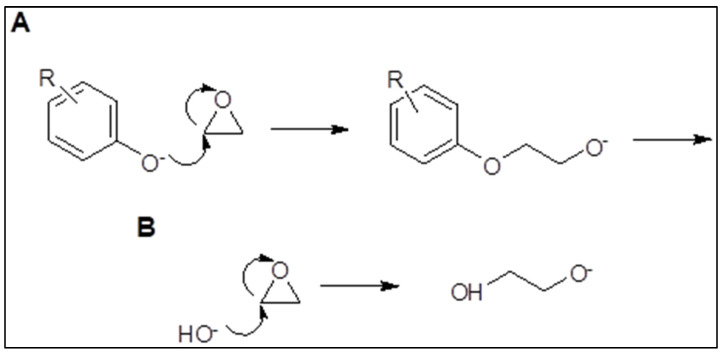
(**A**) Formation of majorethoxylate anion (R = C12H25); (**B**) formation of minor by-product anion.

Synthesis of the employed surfactants was carried out in a lab-scale ethoxylation unit in our laboratories Figure 1. Dodecyl phenol ethoxylate (DPE) and oleic acid ethoxylate (OAE) surfactants were prepared by reacting gaseous ethylene oxide (pressure of 2.5–2.9 psi) with dodecyl phenol (DP) and oleic acid (OA), respectively, at 150–160 °C using KOH catalyst [26] Figure 2 and Figure 3. Polyethylene glycols were formed as by-products and were removed according to the Weibull method [27]. Figure 4 explains the proposed mechanism of the reaction. After the removal of glycols, the obtained surfactants were characterized by FT-IR, and the average number (n) of oxyethylene (OE) units was determined through ^1^H NMR [28].

### 2.4. Thin-Film Composite Nanofiltration Membrane Prepration (TFCNF)

Porous polyvinylidene fluoride support “PVDF” was prepared according to Gao et al. [29] by the phase inversion technique, using the casting solution containing 18 wt% PVDF, 79 wt% DMAc, and 3 wt% phosphoric acids. Dope solutions were kept at 70 °C with stirring for 15 h. The obtained homogeneous solution was left overnight without stirring to eliminate air bubbles. An automatic film applicator cast “PVDF” support at an application speed of 0.05 m s^−1^ using a 250 µm knife set (Sheen 1133 N, Nottingham, UK). The casted membrane was immersed for 10 min in a deionized water bath at room temperature and then kept in another water bath to dry at room temperature for 24 h to be used for thin-film composite nanofiltration membrane (TFCNF) preparation Curcio et al. [30].

Porous PVDF-supported TFCNF membrane was formed by the in-house built system using a dip-coating procedure. For all coating solutions, the polymer content was 4% by weight in chloroform, while the range of the filler loading was 0.01 to 0.25% by weight concerning the polymer weight. PVDF membrane support was dried overnight at room temperature before cutting into (3 cm × 10 cm) coupons. TFCM was prepared by putting the PVDF supports in contact with the coating solutions for 10 min [31].

### 2.5. Experiments of TFCNF Membrane Antiscaling

**Figure 5:**
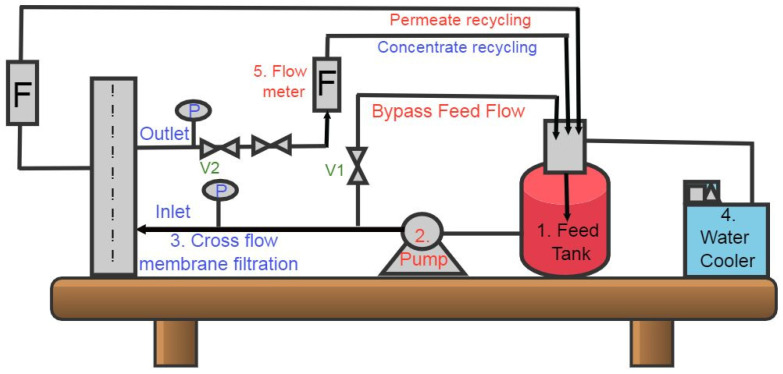
Diagram of membrane scaling experiments process flow.

The permeability and salt rejection of scaled inhibited behaviour of this membrane without and with scale inhibitors were measured by TFCNF membrane anti-scaling experiments. The jar-scale filtration setup was established to perform cross-flow membrane filtration experiments, as reported in Figure 5 [32].

Feed water was pumped from a 20 L tank with a high-pressure pump to the cross-flow membrane filtration cell (CF042, Sterlitech Corporation, Kent, WA, USA).

Figure 5 shows the recirculation of the concentrated water through the circulating water chiller back into the feed tank to control the feedwater temperature. Valves controlled the transmembrane pressure (TMP) in both feed water and concentrate water lines.

Feedwater filtration tests were performed with three initial pH values, “3.4, 7.0, and 10.0”, to determine the effect of the initial feedwater pH on membrane performance. 0.1 N HCl and 0.1 N NaOH were used to adjust the solution pH. Membrane flux was monitored throughout the filtration process and measured by the gravimetric method by weighing the permeability mass collected at certain time intervals. The feed and permeate pH was also monitored and measured with an AD 11 pH meter (ADWA Kft., Europe). The feed and permeate samples were collected continuously throughout the entire filtration test to obtain membrane rejection rates.

The Pure water permeation fluxes (PWP) were measured using Equation (1) at operating conditions 1 L/h (flow rate), 35 °C (temperature), 32 bar (pressure) [32].

(1) $$J_{w}\;\left(PWP\right)=\frac{Q_{p}}{∆P.A}\;$$

where *Jw* is membrane permeation flux (L/m^2^.h.bar), *Q**_p_*** (L/h) is permeate flow rate, Δ*P* (bar) is the trans-membrane pressure, and *A* is the effective membrane area (m^2^).

The effect of membrane scaling on salt rejection was calculated by measuring the conductivity (Digital Conductivity Meter, PCE-PHD 1-ICA) of permeate and feedwater to the scaling experiment end. The (%R) membrane salt rejection was calculated using Equation (2).

(2) $$\%R=\frac{\left(C_{f}-C_{p}\right)}{C_{f}}\times 100\;$$

where *C_f_, C_p_* is the feed water and permeate conductivities.

The experiments were set up and divided into two steps. The first one consisted of conditioning the membranes using ultra-pure water for 1–2 h. In the second step, the feed water was the scaling solution, as the resulting time-dependent flux decline was tracked until it reached the stability of the permeate flux. The membrane flux (J, L/m^2^/h) was calculated using Equation (3) [33]:

(3) $$J=\frac{Q_{p}}{A}$$

where *J* is filtration flux, the normalized flux *J*_N_ is calculated using Equation (4):

(4) $$J_{N}=\frac{J}{J_{0}}$$

where *J*_0_ is an initial filtration flux at a given time.

Before each scaling experiment, the membranes were rinsed thoroughly with deionized water. Experiments were performed with fixed operating conditions under a recycling mode to maintain the feed water conditions as previously mentioned.

### 2.6. Characterizations

The characterizations of the prepared scale inhibitors DPE and OAE were investigated using FT-IR and ^1^HNMR.

Various techniques were applied to characterize the membrane surface before and after scaling experiments; ESEM, FT-IR, XRD. The hydrophilicity was measured by a contact angle device (OCA15Pro, Bruker, Germany). 2 μL water drop was released onto the surface of the membrane mounted in the contact angle device.

Membrane surface scanned using Nova™ NanoSEM 50 Series (FEI Company) for ESEM analysis. FT-IR of the treated membrane was compared with that of the control membrane in spectra of 500–4000 cm^−1^ wavenumber range (Shimadzu FT-IR instrument).

The mineralogical composition and the crystalline nature of the scales deposited on the scaled membrane surface were analyzed via an X-ray diffractometer (XRD, D8 ADVANCE, Bruker, Germany).

Membranes samples evaluated mechanical properties (dog-bone shaped) applied using a universal tensile machine (LFplus 1 kN Lloyd/New York, NY, USA). The uniaxial tensile testing method was performed at a rate of 5 mm/min. Tensile strength (MPa), Young’s modulus (MPa), elongation at fracture (%), and load at maximum load (N) were measured.

Brunauer–Emmett–Teller (BET) surface area analysis was applied to determine the membrane surface area and porosity. Before analyzing, 0.6 g dried membranes samples were degassed under vacuum in a surface area analyzer (AS-3012 AimSizer/Ningbo, China) for 10 h at 65 °C. All istruments used to characterized the prepared scale inhibitors and prepared membrane are found in Egyptian Petroelum Research Institute (EPRI).

## 3. Results and Discussion

### 3.1. Scale Inhibitors and Membrane Characterization

#### 3.1.1. FT-IR of Scale Inhibitors

**Figure 6:**
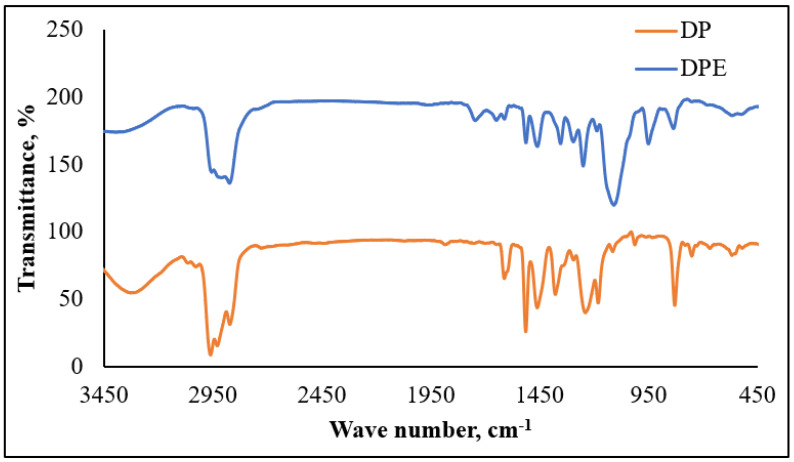
FTIR of dodecyl phenol (DP) and dodecyl phenol ethoxylate (DPE).

**Figure 7:**
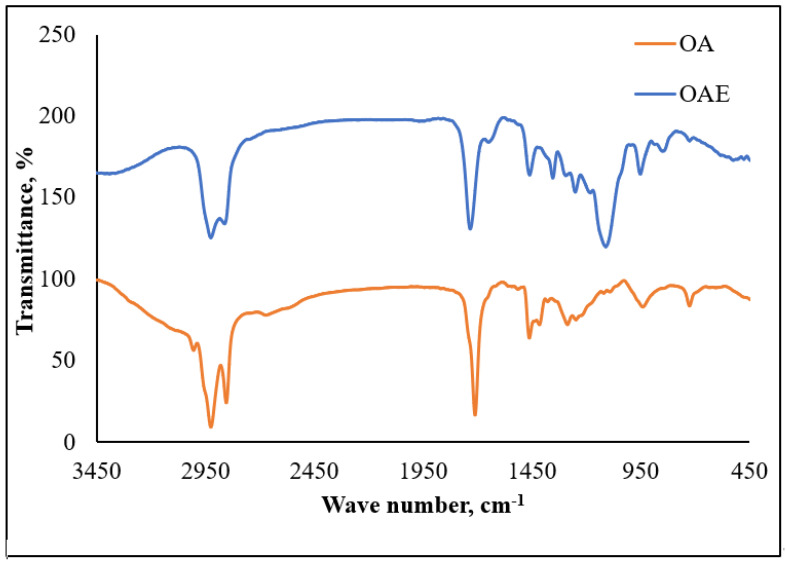
FTIR of oleic acid (OA) and oleic acid ethoxylate (OAE).

The FT-IR spectra (Figure 6 and Figure 7) of the prepared non-ionic compounds showed the most characteristic band of ethoxylate formation, which is a band of ν C-O-C at ~1100 cm^−1^ [26].

#### 3.1.2. H-NMR for Scale Inhibitors

**Figure 8:**
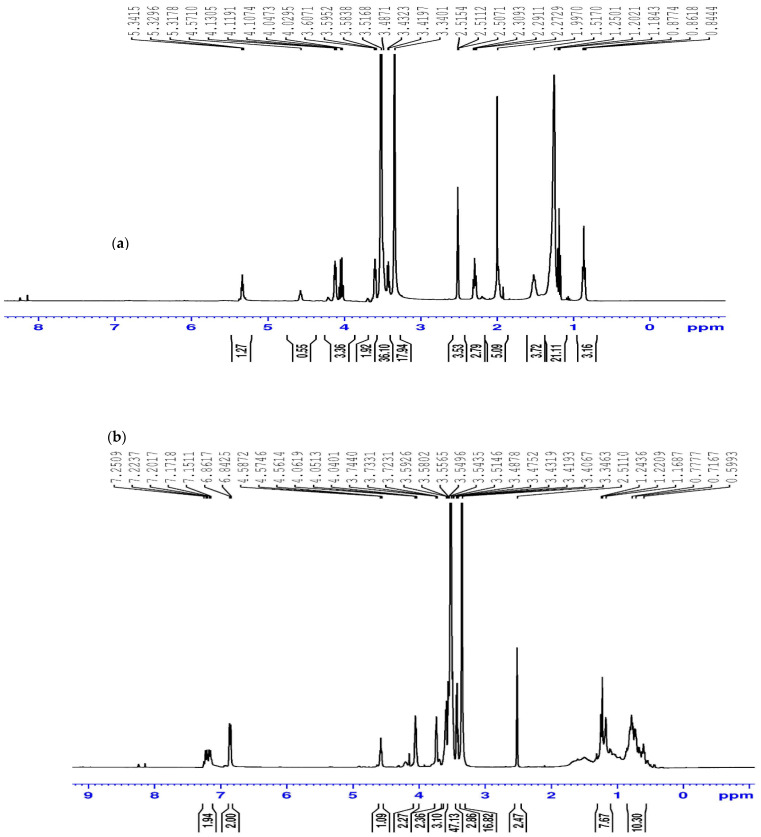
1HNMR of (**a**) Scale inhibitor OAE, (**b**) Scale inhibitor DPE.

^1^H-NMR spectra of the prepared non-ionic surfactants were obtained using Varian Mercury 300 Mz spectrophotometer. DMSO (2.47, 2.51 δ) was the used solvent for the samples. Figure 8a shows the spectra of oleic acid ethoxylate (OAE), which was estimated to have 12.06 as an average number of oxyethylene (OE) units adducted per oleic acid molecule. The spectra show a strong signal at about 3.5, corresponding to the polyoxyethylene chain [28]. The average number of oxyethylene units in the polyoxyethylene chain can be estimated from the relative intensities of the different signals. In ^1^H NMR, the intensity of a signal is proportional to the number of protons creating that signal. Figure 8b explains the spectrum of dodecyl phenol ethoxylate (DPE), which was estimated to have 13 as an average number of oxyethylene (OE) units adducted per dodecyl phenol molecule.

### 3.2. Membrane Scaling Evaluation

#### 3.2.1. Permeability, Salt Rejection, and Permeate Flux Decline

From previous scientific studies [34,35,36], it can be said that increasing the % scales rejection aids in producing a nucleus that forms the calcite scale, which causes a decrease in the permeability of the used nanofiltration membrane in the desalination process. The use of antiscalants such as DPE and OAE in this study for the feeding water increases % calcite scales rejection by chemical reaction and chemosorption process during the first nucleus, which forms the calcite scales on the surface of the NF membrane.

**Figure 9:**
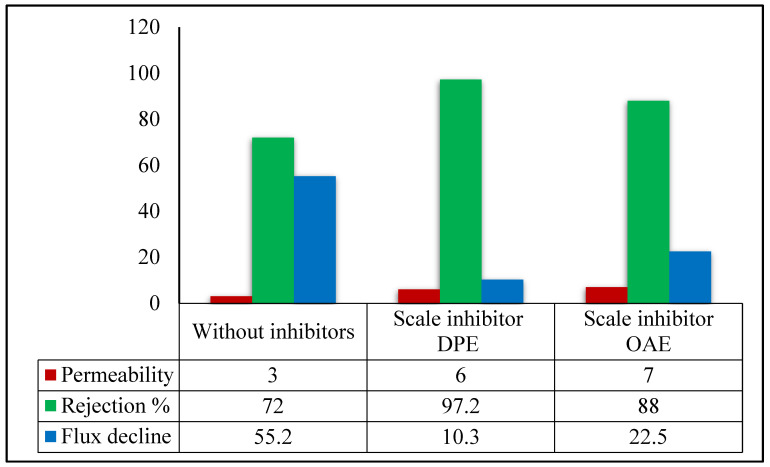
Membrane permeability, % scale rejection, and Flux decline (L/m^2^h) for membrane, without inhibitor; with scale inhibitor DPE; and with scale inhibitor OAE.

Generally, by using DPE and OAE during the PW treatment process, the % scales rejection increases from 72% for blank TFCNF membrane reaching 97.2% and 88% for both DPE and OAE, respectively, as confirmed by data represented in Figure 9. This confirms that the calcite scales are formed in an abnormal brittle structure with a small amount that decreased the number of cleaning cycles of the NF membrane. Additionally, scale inhibitors DPE and OAE can form soluble coordinating stable salts during the treatment process, which positively affects the permeability of the nanofiltration membrane, as shown in Figure 9.

DPE and OAE non-ionic surfactants are expected to increase membrane hydrophilicity. Contact angle tests showed a contact angle (52°) of the blank membrane TFCM membrane, reduced significantly to 26° and 40° for DPE and OAE, respectively, which confirms the higher efficiency of DPE. It was also observed that the normalized permeate flux (L/m^2^.h) decreased to 0.78 ± 0.01; this equals to 55.2% flux decline. On the other hand, the permeate flux decreased by only 10.3% and 22.5% when using DPE and OAE, with no significant impact on the salt rejection capability of the membrane by the end of the scaling experiment. The obtained results of reaching the steady-state conditions (150 min) are shown in Figure 9.

The above results show that scale inhibitor DPE gives a better membrane permeability and high % scales rejection with high scale inhibition efficiency than OAE in the same conditions.

#### 3.2.2. Permeate Flux

**Figure 10:**
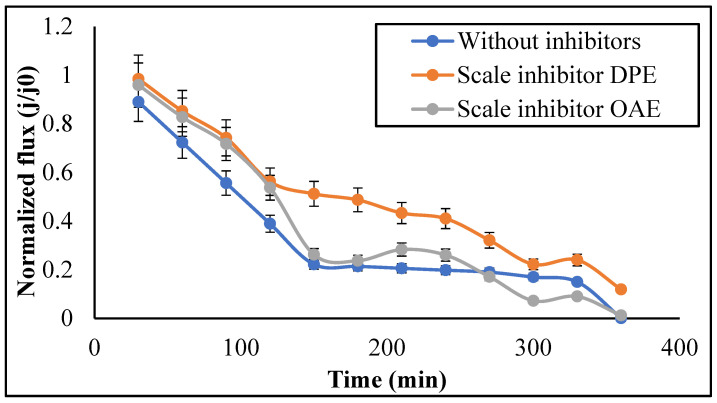
Permeate flux with time for TFCNF membrane without inhibitors, with scale inhibitor DPE, and with scale inhibitor OAE (Flow rate = 1 L/h, temperature = 35 ± 2 °C, pressure = 32 bar).

TFCNF membrane performance against calcite was evaluated by recirculating the prepared calcite solution and monitoring the decrease in flux caused by deposited scales during the experimental time. Figure 10 shows that scaling causes a flux decline over time.

Figure 10 shows the nanofiltration application of oilfield-produced water treatment. The figure shows that the scale inhibitor DPE inhibits transmembrane scaling pressure in the TFCNF membrane. This effect was confirmed by membrane scale characterization using SEM-EDX, FT-IR, and XRD. From this figure, the water flux drops sharply and finally reaches zero after 360 min with and without scale inhibitor OAE feed solution. In contrast, the water flux drop was significantly reduced during the 350 min continuous scaling test with the DPE scale inhibitor feed solution. Accordingly, by comparing the injection of the scale inhibitor OAE and DPE feed solutions with the same initial water flux, the lower water flux of the DPE feed solution for scale inhibitor is also lower during the membrane scaling test. These experiments demonstrate that the scale inhibitor DPE has been shown to have a higher enhancing effect on TFCNF filtration ability.

#### 3.2.3. Effect of pH Value on Calcite Scale Formation

**Figure 11:**
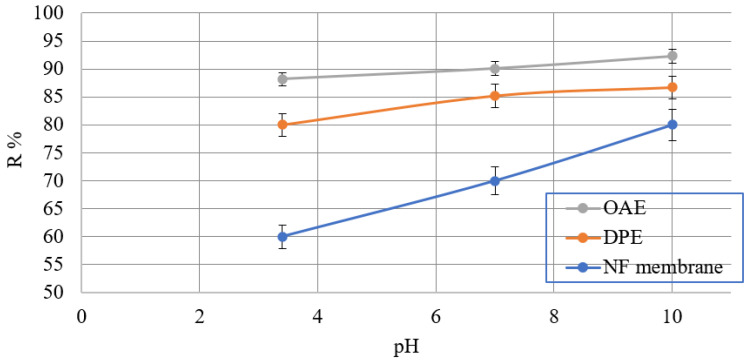
The rejection rates of calcite ions by NF membrane throughout the filtration tests at different initial feed water pH (Flow rate = 1 L/h, temperature = 35 ± 2 °C, pressure = 32 bar).

A series of experiments were performed with the feed water at different pH to investigate the effect of the initial pH of the feed water on the rejection rates of calcite by the NF membranes in Figure 11. It is clear from the figure that the rejection rate of calcite scales by the NF membrane was very low by the NF membrane. Only calcite scales were added to the feed tank (initial pH: 3.4). Initially, the rejection rate of calcite scales was about 60%. Then it started to increase with increasing the PH value in the feed solution and finally stabilized at about 80%. It was confirmed by repeating the experiment, similar results were obtained. However, when the pH of the feed water started to increase up to 10.0 by adding NaOH solution, the rate of calcite scales rejection by the NF membrane increased to 92.3, 86.7, 80% for OAE, DPE, and NF membrane. We can say that increasing the feed water’s pH can enhance the NF membrane’s rejection performance against calcite scales in an aqueous solution. So, there was an effective change observed between the two-scale inhibitors (DPE and OAE) feed solutions, more than the NF membrane without them.

### 3.3. Simulation of CaCO_3_ Scaled Membrane

Testing the scale layers formed on the thin-film composite membrane after the scaling experiment is important to explain the enhancing effect of the two inhibitors on the nanofiltration action and highlight the progress of the layering process. ESEM, XRD, and FTIR techniques were used.

#### 3.3.1. ESEM for Inhibited Scaled TFCNF Membrane after Injection the Scale Inhibitors

**Figure 12:**
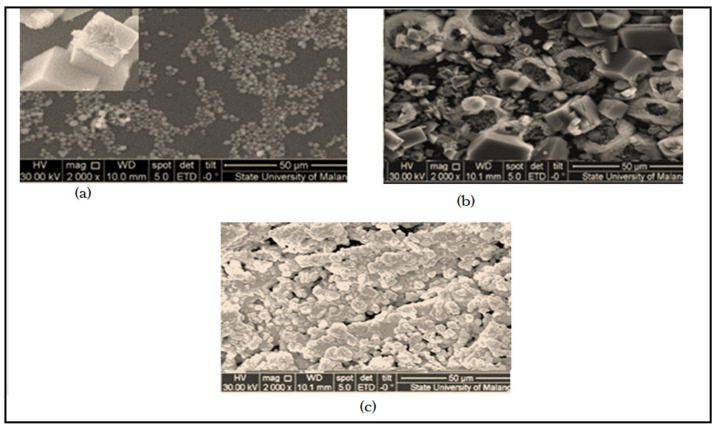
(**a**) ESEM of CaCO_3_ scaled membrane without scale inhibitors injection; (**b**) with scale inhibitor DPE injection, and (**c**) with scale inhibitor OAE injection.

Environmental scanning electron microscope (ESEM) images of a calcite scaled membrane without scale inhibitors injection; with scale inhibitors, DPE, and OAE injection are observed in Figure 12a–c. In all cases, there is a common observation that CaCO_3_ small crystals are formed. Calcite single crystal morphology shows a well-developed rhombohedral shape, characterized by straight and sharp edges (Figure 12a).

In the absence of a scale inhibitor, calcite crystals have an increased size, with a distorted shape showing rounded contours on the face perimeters (Figure 12a). Only the part facing the incoming flow appears in the well-established growth of rhombohedral calcite.

On the other hand, with injection scale inhibitors DPE, and OAE, although macroscopically, the CaCO_3_ scale deposits show a uniform distribution on the TFCNF membrane. The CaCO_3_ crystals are close to the exit in larger size compared with crystals close to the entry. This may be due to increasing concentration polarization as shown in Figure 12b,c, respectively.

#### 3.3.2. XRD Spectra of the Inhibited ScaledTFCNF Membrane

**Figure 13:**
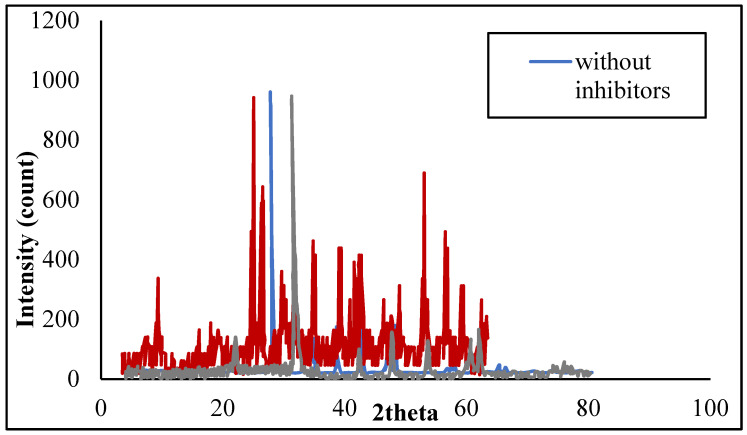
XRD of inhibited scaled TFCNF membrane without inhibitor; with scale inhibitor DPE injection; and with scale inhibitor OAE injection.

XRD reveals important information about the inhibited scaled crystal structure, size, shape, and nature. Figure 13 shows XRD patterns of TFCNF membrane with and without scale inhibitors. It is clear from Figure 13 that for the scaled TFCNF membrane without inhibitors, two weak and broad peaks around 25 and 45 were observed, which are characterized for calcite scale. The spectra showed the calcite peak disappeared in the presence of the antiscalant.

#### 3.3.3. FTIR Spectra of CaCO_3_ Scaled Membrane

Different sites were sampled throughout the membrane surfaces and analyzed by FTIR to detect the nature of scales deposits.

**Figure 14:**
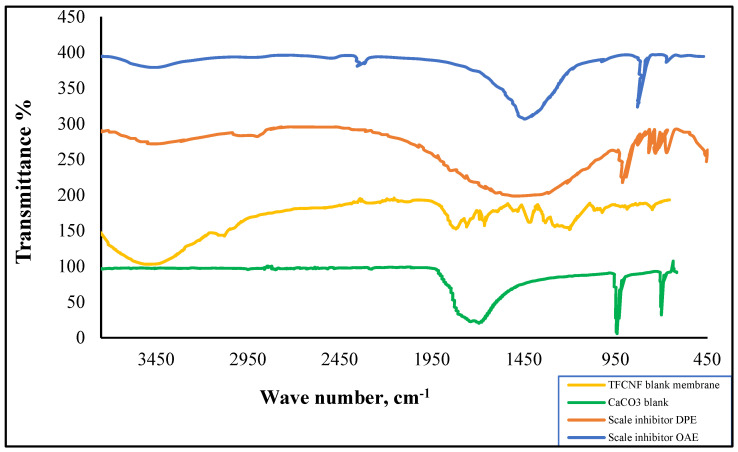
FTIR of TFCNF membrane (**a**) blank membrane; (**b**) scaled membrane without inhibitors; (**c**) with S.I. DPE injection; and (**d**) with S.I. OAE injection.

In the current work, similar to the literature [37,38,39], the FTIR spectra suggested that the strong bands centered around 1140 cm^−1^ splitting into two components at around 1146 cm^−1^, 1116 cm^−1^, and 669 cm^−1^, 662 cm^−1^ represents stretching and bending modes of CO_3_ from pure calcite. An extensive scale layer formation is seen throughout nearly the entire membrane surface in all samples (Figure 14). In contrast, in DPE and OAE injection in feed-produced water, the calcite can be detected only towards the highly saturated zone in the flow exit region.

Calcite peaks at 1400 cm^−1^, 1020 cm^−1^, 1500 cm^−1^, and 1650 cm^−1^ attribute to polymorph of calcite precipitates, detected in both the highly saturated zone and throughout the filtration membrane surface.

#### 3.3.4. Mechanism of Membrane Scaling Control with S.I. DPE and OAE

**Figure 15:**
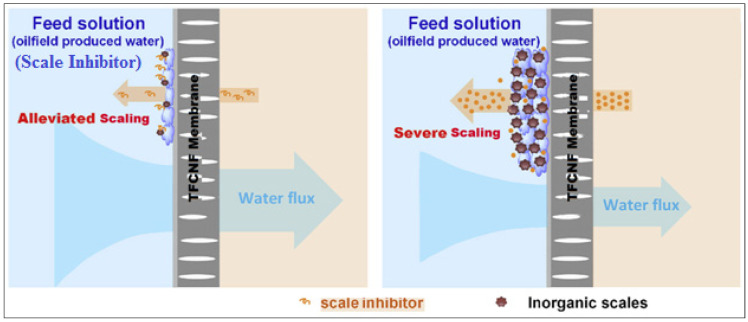
TFCNF membrane mechanism for reduced calcite with and without scale inhibitors in oilfield produced water.

As the schematic diagram (Figure 15) illustrates, the higher scaling-control efficiency using scale inhibitor DPE in feed solution can be due to the higher DPE concentrated near the filtration membrane surface. It is clear from Figure 15 that with the injection of scale inhibitors into the feed solution, DPE is chelated by Ca^2+^ in the bulk solution, resulting in the reduction of DPE concentration adjacent to the membrane surface.

Alternatively, with the DPE feed solution employed in the NF process, the diffusion of DPE draw solute from draw solution into the feed solution is continuous. Additionally, the concentration near the membrane surface is much higher than that in the bulk feed solution, which guarantees a better anti-scaling effect on the membrane. In addition, the leaked S.I.DPE draw solute adsorbed on the surface of the formed calcite crystals can further restrain the calcite crystal growth on the membrane surface effectively. Moreover, DPE can also cause the lattice distortion of calcite crystals [37], resulting in loose scales on the membrane surface, which a physical cleaning can easily wash away.

The improved anti-scaling performance of the TFCNF membrane rises from better hydrophilicity and thus surface smoothness. Soanenergy barrier would develop against calcite surface nucleation or deposition on the membrane surface. Moreover, the repulsive forces between the negative charges of scale inhibitors and calcite ions may have played an important role in discouraging fouling on the membrane [38].

### 3.4. Comparison of OAE and DPE with Other Scale Inhibitors

In Table 2, our results are compared with the previous work summarizing the efficiency of scale inhibitors to prove the effectiveness of OAE and DPE as calcite scale inhibitors. Their effectiveness was compared to the scale inhibitors as salt rejection %. It is clear from this comparison that the scale inhibitor DPE can be used as an effective scale inhibitor during the anti-scaling membrane process, as it has a high percentage of rejected salts %. Features of different S.Is., such as structure, functional groups, and surface areas, are attributed to differences in scale inhibition ability [39].

## 4. Conclusions

This paper aims to reduce the calcium carbonate scales from PW precipitated in oilfield production pipelines to enhance oil production. TFCNF membrane was prepared and its function was enhanced via the injection of two new scale inhibitors (DPE and OAE). Using scale inhibitors, filtration experiments revealed that the flux decline is only 15% in the presence of the scale inhibitors, whereas the flux decline in the TFCNF membrane is 22%. SEM shows no precipitates were observed on the membrane.

According to the results of FT-IR and XRD measurements performed on the scaled membrane, the presence of precipitates was restricted to only highly saturated zones (i.e., zones near the end of the water flow channel) on the membrane. The injection of scale inhibitors DPE into the feeding solution can effectively reduce calcite scales’ formation on the surface of the TFCNF membrane. This is attributed to the fact that it has excellent scale inhibiting ability as well as unique diffusion. This research may result in a practical strategy for membrane scaling control in the NF application of produced water treatment and a comprehensive understanding of the subject.

## Figures and Tables

**Table 1:** Composition of synthetic produced water (PW).

Concentration of Ions (mg/L)		Density(g/cc)	1
Na^+^	2000	pH	7.81
Cl^−^	3437.46	TDS	6447.55
Ca^2+^	400	Salinity	6010
HCO_3_^−^	610	Alkalinity as HCO_3_^−^ (mg/L)	599.53

**Table 2:** Comparison of OAE and DPE efficiency with some calcite scale inhibitors of previous studies.

Antiscalant	Salt Rejection (%)	Ref.
AA-APEC	96%	[40]
CM-QAOC	70.2%	[41]
Palm leaves extract	89.7%	[42]
PASP/Cs	92%	[43]
PAA	82.7%	[44]
AA-APEC	83.6%	[44]
CG	91%	[45]
OAE	88	This study
DPE	97.2	This study

## Abbreviations

**S.I.**	**Scale Inhibitor**
NF	Nanoafiltration
TFCNF	Thin-film composite nanofiltration membrane
FT-IR	Forior transform IR
XRD	X-ray diffraction
ESEM	Environmental scanning electron
DP	Dodecyl phenol
DPE	Dodecyl phenol ethoxylate
OA	Oleic acid
OAE	Oleic acid ethoxylate
PW	Produced water
Na_2_CO_3_	Sodium carbonate
CaCl_2_	Calcium chloride
DMAc	Dimethyl-acetamide
DCM	Dichloromethane
OcA	Octylamine
OcDA	Octadecylamine
PVDF	Poly-vinylidene fluoride
AA-APEC	Acrylic acid allylpolyethoxy carboxylate
CM-QAOC	Carboxymethyl quaternary ammonium oligochitosan
PASP/Cs	Polyaspartic acid/chitosan
PAA	Polyacrylic acid
CG	Chitosan biguanidine hydrochloride
